# Fistulotomy versus standard cannulation as the primary technique for all patients undergoing ERCP with a native papilla: a protocol for a single center randomized controlled trial

**DOI:** 10.1186/s13063-022-06084-4

**Published:** 2022-02-16

**Authors:** Lawrence Hookey, Mandip Rai, Robert Bechara

**Affiliations:** Department of Medicine, Division of Gastroenterology, Queen’s University, Kingston, Ontario Canada

**Keywords:** ERCP, Pancreatitis, Post-ERCP pancreatitis

## Abstract

**Background:**

Endoscopic retrograde cholangiopancreatography (ERCP) is an indispensable procedure for the management of pancreaticobiliary diseases. Post-ERCP pancreatitis (PEP) is the most common serious adverse event. One risk factor of PEP is difficulty achieving biliary access. The conventional ERCP technique involves the cannulation of the bile duct via the ampulla of Vater, followed by sphincter incision using electrocautery. Conventionally, if the standard method fails then, precut techniques have been utilized as an alternative means of gaining biliary access. The needle-knife fistulotomy (NKF) technique involves identifying the intra-duodenal segment of the bile duct and uses a needle knife to incise directly into the bile duct. This is done above and away from the natural office, thus minimizing thermal damage which may result in PEP. Our recent prospective study of 50 patients demonstrated the safety and feasibility of the NKF precut technique as a primary means of gaining biliary access. The next step is to conduct a randomized controlled trial to compare the efficacy and safety of initial NKF to the standard cannulation in a non-selective patient population undergoing ERCP.

**Methods:**

A randomized control trial of 538 consecutive, non-selective patients with pancreaticobiliary disease undergoing ERCP at a tertiary care center in Kingston, Ontario, Canada, will be conducted. Patients will be randomized to one of two treatment groups, standard cannulation or NKF. The primary outcome of the study will be the incidence of PEP. Secondary outcomes will include rate of successful cannulation of the common bile duct (CBD), time to successful cannulation, total procedure time, difficulty of cannulation, and incidence of complications.

**Discussion:**

This RCT will yield important answers regarding the efficacy and safety of initial NKF to the standard cannulation in a non-selective patient population undergoing ERCP. The results of our study could alter ERCP practices and outcomes if NKF is shown to reduce PEP risk.

**Trial registration:**

ClinicalTrials.govNCT04559867. Registered on September 23, 2020

## Background

Since its inception in 1968, endoscopic retrograde cholangiopancreatography (ERCP) has become an indispensable procedure for the management of pancreaticobiliary diseases [1]. Six years later, the first endoscopic sphincterotomies were completed by Kawai and Claussen and it is now used for indications including, but not limited to, stone removal from the common bile duct (CBD), management of papillary stenosis, or type 1 sphincter of Oddi (SOD) dysfunction and to facilitate the delivery of pancreaticobiliary therapy [2, 3]. The procedure, however, continues to result in significant morbidity in a small but not insignificant number of patients. Post-ERCP pancreatitis (PEP) is the major source of this, affecting between 5 and 7% of patients undergoing the procedure [2]. For 10% of patients who experience PEP, it can be severe and even fatal [3].

One risk factor of PEP is difficulty achieving biliary access. Factors include but are not limited to increased time to cannulation, increased number of cannulation attempts, and pancreatic duct cannulations. Although medical interventions such as intravenous volume expansion, pancreatic protease inhibitors, and NSAIDs have shown promise or even benefit, the risk of PEP persists [4–11]. Therefore, any intervention or technique that can minimize or even eliminate post-ERCP pancreatitis is highly desired.

The conventional technique involves the cannulation of the bile duct via the ampulla of Vater, followed by sphincter incision using electrocautery [12, 13]. This opens the distal end of the bile duct, allowing easier access for stents and removal of larger objects such as stones. It has been reported, however, that standard access techniques fail between 5 and 10% of the time [2]. If the standard method fails, then conventionally, precut techniques have been utilized as an alternative means of gaining biliary access. There are various precut techniques used to gain access to the common bile duct (CBD), which include needle-knife fistulotomy (NKF), needle-knife sphincterotomy (NKS), and trans-pancreatic precut sphincterotomy (TPS) [14, 15].

Needle-knife fistulotomy involves identifying the intra-duodenal segment of the bile duct and uses a needle-knife to incise directly into the bile duct. The fistula is away from the native orifice of the papilla. NKS involves using a needle knife to cut starting at the native orifice and incising upwards to expose the bile duct opening. A TPS involves incising the pancreatic sphincter, which lies adjacent to the biliary sphincter (within the native orifice), to expose the biliary opening.

Numerous studies have demonstrated that adverse event rates are comparable between methods when precut sphincterotomy is conducted by an expert endoscopist [16] and that moving to precut techniques earlier in the procedure can be associated with fewer adverse events [17–19]. With respect to which of the three techniques to employ, the NKF theoretically offers the lowest risk of pancreatitis as the incision is performed directly into the intra-duodenal segment of the bile duct, minimizing any contact or thermal damage to the pancreatic duct. This theory is supported by the results in the series by Jin et al., who observed a 0% incidence of pancreatitis using the NKF method as the initial technique for cannulation in high-risk patients [20]. Additionally, our recent prospective study of 50 patients demonstrated the safety and feasibility of the NKF precut technique as a primary means of gaining biliary access [21]. Without a control group, however, our trial could not compare the current results to the standard cannulation technique.

The next logical step then is to conduct a randomized controlled trial (RCT) to compare the efficacy and safety of initial NKF to the standard cannulation in a non-selective patient population undergoing ERCP. The objective of this study is to add to the literature of ERCP that assesses NKF as the initial method of gaining biliary access. The primary outcome of the study will be the incidence of PEP. Our hypothesis is that NKF will have a lower PEP rate than standard cannulation.

## Methods

### Study design and setting

**Fig. 1:**
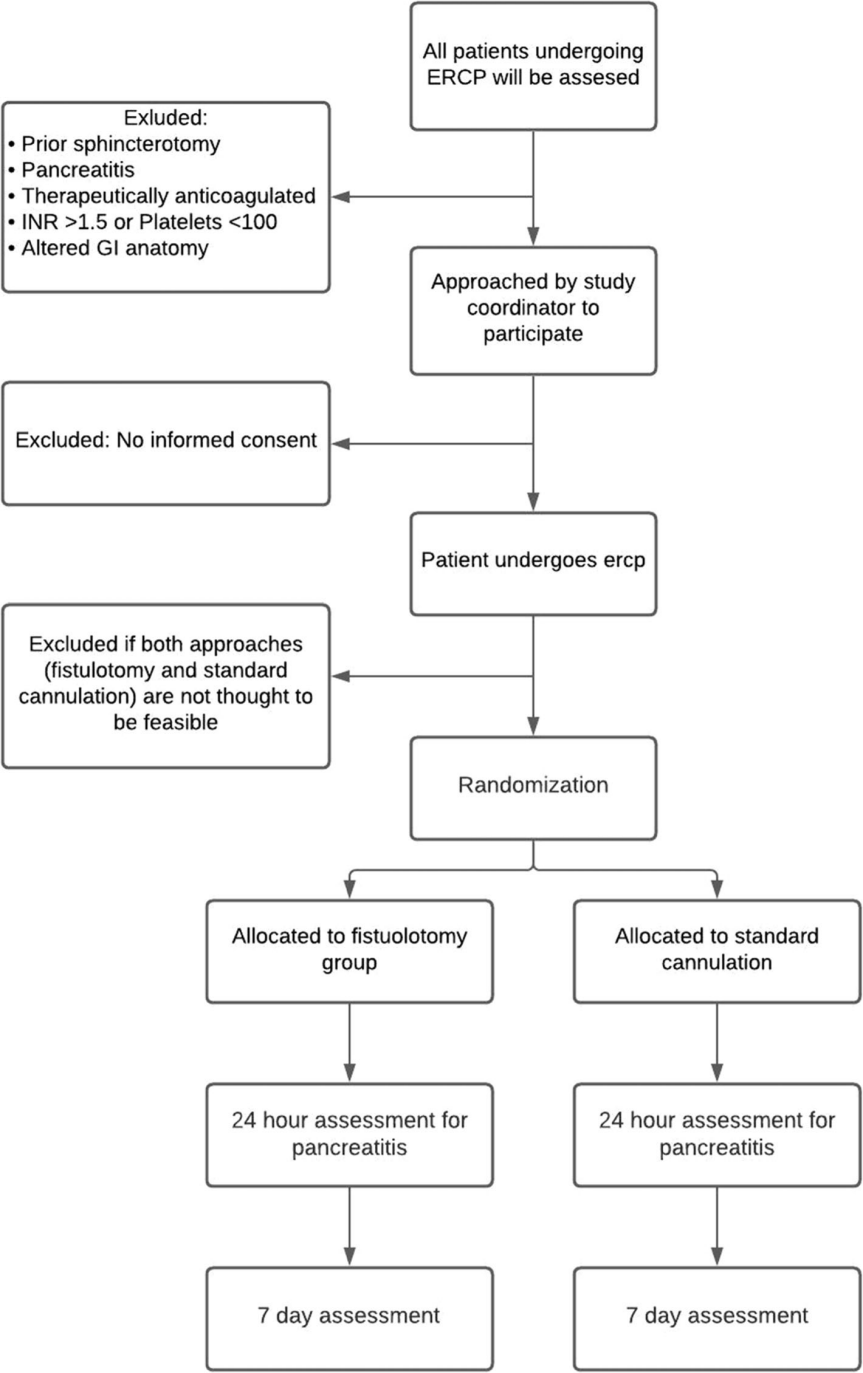
Flow of participants

This protocol (version 2.4) was written and reported according to the Standard Protocol Items: Recommendations for Interventional Trials (SPIRIT) recommendations [22], and a SPIRIT checklist is provided in the supplemental materials. This is a randomized, controlled, superiority trial comparing NKF to standard sphincterotomy for the primary endpoint of PEP. Secondary outcomes will further compare the safety and efficacy of the two methods. The study will take place at a single, tertiary ERCP referral center in Kingston, Ontario, Canada. All procedure will be performed by two experienced endoscopists having performed over 1000 ERCPs. The flow of participants is summarized in Fig. 1.

### Outcome measurements and definitions

1. The primary outcome of the study will be the incidence of PEP, defined as abdominal pain consistent with acute pancreatitis and an elevation greater than three times the upper limit of normal of serum lipase levels [23].
2. The secondary outcomes of the study include:
  - Rate of successful cannulation of the CBD, with technical success determined by a cholangiogram;
  - Inspection time; defined as time from visualization of ampulla to appearance of needle knife or sphincterotome;
  - Time to successful cannulation, measured as follows:
    i. Standard cannulation group (group I): time of endoscopic visualization of the sphincterotome on the screen to contact with the papilla orifice;
    ii. NKF group (group II): time of endoscopic visualization of the metal point of the needle knife on the screen to successful CBD cannulation as evident by the cholangiogram or wire advancement into the CBD;
  - Total procedure time, measured for completed procedures from the time of esophageal intubation to the time of scope withdrawal from the patient mouth,
  - Ampullary morphology—cannulation success and pancreatitis rates
  - Difficulty of cannulation, graded on a 3-point scale, based on the operator’s subjective opinion (easy, moderately difficult, very difficult), and,
  - Incidence of complications graded and defined by Cotton et al. [20], notably, intra-procedural bleeding that required intervention (cautery, clips, or injection of epinephrine), delayed or sustained bleeding requiring transfusion or repeat endoscopy and immediate or delayed perforation.

### Inclusion/exclusion criteria

Five hundred thirty-eight consecutive patients meeting inclusion/exclusion criteria, undergoing biliary ERCP by two expert endoscopists, will be considered for inclusion.

*Inclusion criteria*:

1. Patients, ages > 18, with an intact ampulla undergoing ERCP for therapeutic purposes who can provide informed consent. This includes patients who have confirmed choledocholithiasis on imaging and those who have a high suspicion of it based on imaging and lab values. Patients with and without a high suspicion for cholangitis will be eligible for the study. Other indications include the following: other benign biliary duct diseases including strictures, primary sclerosing cholangitis, and Mirizzi syndrome requiring biliary decompression. Furthermore, patients with suspected diagnosis of biliary leak following cholecystectomy will also be considered for enrollment in this study.
2. Ability to read and understand the English language,
3. Ability to follow-up in a reliable manner.

*Exclusion criteria*:

1. Bleeding disorder (Von Willebrand disorder, platelet count < 100,000, or INR > 1.5),
2. Therapeutic level anticoagulation with low molecular weight heparin (LMWH), warfarin, or a direct-acting oral anticoagulant (DOAC),
3. P2Y12 inhibitors not held for 5 days prior to the procedure,
4. Prior biliary sphincterotomy,
5. Concurrent pancreatitis (with inability to tolerate oral intake and requiring pain management),
6. Altered upper GI tract anatomy (e.g., prior gastric bypass surgery such as Roux-en-Y or Billroth 2 gastrojejunostomy),
7. Inability to achieve adequate sedation,
8. Evidence of malignant infiltration of the ampulla or peri-ampullary area,
9. NFK or standard cannulation is thought not to be feasible.
10. Pregnancy,
11. Operator inability to identify and access the intra-duodenal portion of the bile duct,
12. Presumptive diagnosis of sphincter of Oddi dysfunction,
13. Inability to access intraduodenal segment due to altered anatomy (e.g., ampulla within deep diverticulum),
14. Requirement for pancreatogram or pancreatic intervention,
15. Inability to provide informed consent.

*Recruitment plan:*

Three groups of patients will be approached for consideration of inclusion in this study:

1. Inpatients at Kingston Health Sciences Centre (KHSC) requiring an ERCP,
2. Clinic patients, for whom an ERCP will be scheduled at a future date,
3. Patients admitted from outside hospitals or out-patients requiring an ERCP.

As per our case series, the first two groups (in-patients and clinic patients) will be identified by the treating gastroenterologist. If the patient is a candidate for the study, the study coordinator will approach the patient and inform them about the background of the study, the risks, the benefits, and answer questions. The signing of the consent form will take place in either the in-patient’s room, a clinic room, or the endoscopy preparation area before the procedure.

Recruitment of the third group of patients (coming directly to KHSC for the ERCP from an outside hospital) will be conducted in a different manner. When the study doctor accepts the referral for the ERCP, the study coordinator will be notified. The study coordinator will meet the patient after they have arrived at KHSC for their procedure and explain the nature of the study, the risks, and benefits, and answer any questions. If the patient is interested, written informed consent will then be obtained before the procedure.

Due to the evolution of the clinical decisions that are made with respect to the ERCP procedure, it may not always be feasible to temporally separate the process of informing a potential participant and consenting a participant. In these exigent circumstances, patients will still be offered the option to participate.

If a participant has formally requested not to participate in research studies, or to be contacted for research purposes/inquires, as indicated in the electronic medical record, they will not be approached for recruitment by the study coordinator.

### Procedure

After obtaining consent, baseline patient characteristics (age, sex, indication for ERCP, preprocedure blood work including ALP, bilirubin, INR, hemoglobin and lipase) and the suspected diagnosis will be documented. The ERCP procedure will follow standard practice for patient preparation, sedation, and intubation of the duodenum. Patients will not be blinded to their treatment group. Patients will receive sedation in the form of midazolam, diazepam, fentanyl, and/or dimenhydrinate. Diclofenac will be administered per rectum at the conclusion of the procedure.

Access to the CBD will be attempted via either NKF or a standard sphincterotome. Upon identification of the ampulla, the operator will evaluate the feasibility of performing both the needle-knife fistulotomy and standard cannulation. If both approaches are feasible, then participants will be randomized with a 1:1 allocation to one of two treatment groups using a central online randomization program (Randomizer for Clinical Trials app, Medsharing, France) and block randomization. The block sizes will not be disclosed to ensure concealment. Should the operator determine that they are unable to safely perform one of the approaches, then the participant will not be randomized. Allocation concealment will be ensured, as the online randomization program will not reveal the treatment group until the endoscopist confirms both approaches are feasible.

To perform the NKF, the ampulla will be identified and closely examined to accurately delineate the infundibulum. A needle knife (Needlecut 3 V, Olympus Medical Canada, Markham, Ontario) will be used to make a 2–3-mm incision in the mid to proximal third of the vertical axis in the intra-duodenal segment of the bile duct. The needle will be used to cut through the mucosa with intermittent examination for the muscular ampullary complex and subsequent penetration into the bile duct usually signaled by bile flow. When bile is seen, the fistula will be gently probed with a guidewire until the CBD is cannulated. Cannulation of the CBD will be confirmed with proximal advancement of the guidewire and contrast injection with cholangiography. If the findings on the cholangiogram indicate that further interventions are necessary, then the fistulotomy site will be extended with the use of standard sphincterotome or balloon sphincteroplasty as per operator. Further interventions include placement of metal or plastic biliary stents, brushing of the ducts for cytology, balloon dilation of strictures, intraductal cholangioscopy basket retrieval of stones, and balloon sweeping of the CBD.

The standard cannulation will be performed using a traditional sphincterotome (Clever-cut 3 V, Olympus Medical Canada, Markham, Ontario) with access gained to the biliary system via the native orifice (group I).

The operator will switch approaches under the following circumstances: [1] the patient was randomized to the sphincterotomy group (group I), but cannulation is not achieved in 10 min or [2] the patient was randomized to the NKF group (group II), but the operator is unable to achieve biliary access and feels they cannot safely cut further.

### Initial data collection and data management

Pre-procedure demographics, as well as lab results of liver enzymes, total bilirubin, lipase, international normalized ratio (INR), hemoglobin, platelets, and the white blood cell count will be recorded. The physician will document any abdominal pain and/or tenderness, both for severity and location. The suspected diagnosis will be documented along with radiologic findings.

Intra-procedure data collection will include total sedation used, sedation start time, endoscope insertion time, visualization of the ampulla, visualization of needle knife or sphincterotome on the screen, time of the sphincterotome making contact with papilla orifice, time of successful cannulation of the bile duct, time for remaining interventions, time of scope out of mouth, and administration of diclofenac. Each procedure will be recorded for verification purposes.

Other data recorded includes the following: ampullary morphology, difficulty of cannulation, normality/abnormality of the CBD, CBD stent placement, cannulation of the pancreas, pancreatic stent placement, pancreatic contrast injection, and any intraprocedural complications.

Post-procedure data will include assessment for pain/ pancreatitis at 24 h post-procedure. If the subject is an inpatient, this will include clinical assessment. If the subject is an out-patient, then they will be contacted by phone and questioned regarding abdominal pain. Should the subject report abdominal pain consistent with pancreatitis, they will be advised to come to the nearest emergency room for assessment as per current standard of care. A post-procedure day 7 phone call will also be scheduled, and subjects will be questioned regarding abdominal pain. Any post-procedure complications will be recorded as previously outlined. Outcome assessors will not be blinded to the treatment group.

Data will be inputted electronically by a full-time research assistant and stored and managed on a secure encrypted program.

### Statistics and sample size

For the sample size calculation, we assume a PEP incidence of 7% with standard cannulation and expect a decrease to 2% in the fistulotomy group. Therefore, 538 patients will be needed (269 in each group) to assess the hypothesis with 80% power and an alpha of 0.05%.

The primary outcome will be assessed in an intention to treat principle. In addition, we plan to report on the number of patients that switched to NKF or standard cannulation following randomization. Primary and secondary outcomes will be assessed using regression methods. A log-binomial model will be used to generate an adjusted relative risk with 95% confidence intervals for the primary outcome. Linear regression models will be used for the secondary outcomes where appropriate. Normally distributed variables are presented as means with standard deviation (SD) and compared using Student’s *t*-test. Non-normally distributed variables are presented as medians with interquartile range (IQR) and compared with the Mann-Whitney-Wilcoxon test. Based on our pilot study, lost to follow-up is not expected to be an issue [21]. For less than 5% loss to follow-up, a complete case analysis will be done. For more than 5% loss to follow-up, multiple imputation and complete case sensitivity analyses (all combinations of the extremes of what could have occurred) will be done. Multiple imputation will be done in SPSS statistical software with all variables missing listed and five imputed datasets will be created.

### Confidentiality

All information obtained during this study is strictly confidential and subject’s anonymity will be protected at all times. Subjects will be identified by a study number and will not be identified in any publication or reports. The information that is collected for the study will be kept in a locked and secure area by the primary investigator for 25 years.

Only the study team or the people or groups listed below will be allowed to look at the study records:

- Representatives of the Research Ethics Board at the Queen’s University Health Sciences and Affiliated Teaching Hospitals Research Ethics Board
- Kingston Health Sciences Centre, to oversee the ethical conduct of research at this location

### Safety reporting

The risks of NKF are similar to standard technique for ERCP. It is possible that there are unforeseeable risks associated with this procedure.

The reporting investigator will complete the serious adverse events (SAE) report, including date of event, admissions, diagnosis details, and date of discharge. SAEs will be reported to the Research Ethics Board (REB), where in the opinion of the principal investigator (PI) the event was:

- “related”: that is, it resulted from administration of any of the research procedures;
  and
- “unexpected”: that is, the type of event is not listed in the protocol as an expected occurrence.

Reports of SAEs that are both related and unexpected will be submitted to the REB within 2 days of the PI becoming aware of the event.

A data safety monitoring board comprised of three gastroenterologists independent to the study with no competing interests will be reviewing the adverse events and enrollment every 50 cases. The study will be prematurely terminated should there be a significantly increased complication rate associated with needle-knife technique (over 20% of cases) or should successful cannulation of the CBD be less than 80%.

### Study integrity, ethics, and registration

No industry-related funding has been received to support this study or compensate study investigators. The study has received full ethics approval from the Queen’s University Health Sciences and Affiliated Teaching Hospitals Research Ethics Board (DMED-2336-20). All methods will be performed in accordance with the relevant guidelines and regulations. The study is registered on clinicaltrials.gov NCT04559867 (23/09/2020). Any modification to the protocol which may impact the conduct of the study and potential benefit of the patient or may affect patient safety, including changes of study objectives, study design, patient population, sample sizes, and study procedure, will require a formal amendment of the protocol. The ethics committee/IRB will be notified. Dissemination of study results is planned through publication at medical conference and in peer-reviewed journals.

The study is being funded by the Southeastern academic medical organization innovation grant. The funding source had no role in the design of this study and will not have any role during its execution, analyses, interpretation of the data, or decision to submit results.

## Discussion

This RCT will assess the efficacy and safety of initial NNF to standard cannulation in a non-selective patient population undergoing ERCP. The primary outcome to be examined is the incidence of PEP. ERCP is a commonly performed procedure and PEP is the most common serious adverse event. The pathophysiology of PEP is not entirely clear with etiology likely involving a combination of chemical, thermal, mechanical, hydrostatic, enzymatic, allergic, and microbiological insults that result from papillary instrumentation and/or hydrostatic injury from the overfilling of the pancreatic duct with contrast material [24]. NKF theoretically offers the lowest risk of pancreatitis as the incision is performed superior to the papillary orifice directly into the intra-duodenal segment of the bile duct under direct visualization, minimizing any contact or thermal damage to the pancreatic duct.

In our pilot study, NNF was shown to be at least as safe as the traditional access technique with a sphincterotome [21]. A recent study by Jang et al. showed that NNF was an effective and safe procedure to gain primary biliary access with a lower PEP rate [25]. However, this study only included patients at high risk for PEP and prophylactic NSAIDs were not used in this study. The European Society for Gastrointestinal Endoscopy recommends rectal indomethacin or diclofenac for all patients undergoing ERCP without contraindications [26]. Diclofenac will be administered per rectum at the conclusion of the procedure in our study.

Recruitment for this study is planned to start in the late fall of 2020 and expected to conclude by the end of 2022. Loss to follow-up is expected to be low, given that PEP occurs within days of a procedure and the planned assessment at 24 h and 7 days post-procedure. This will be done in person if the patient is admitted to hospital or via phone call from a research assistant if the patient is not admitted.

In conclusion, this RCT will yield important answers regarding the efficacy and safety of initial NNF to the standard sphincterotomy in a non-selective patient population undergoing ERCP. The results of our study could alter ERCP practices and outcomes if NNF is shown to reduce PEP risk.

## Trial status

Recruitment started December 1, 2020. This is protocol version 1.0. Approximate date for recruitment completion is December 2022. This trial was registered on clinicaltrials.gov (NCT04559867) on September 23, 2020. https://clinicaltrials.gov/ct2/show/NCT04559867?term=bechara&draw=2&rank=2

## Data Availability

Not applicable.

## Abbreviations

ERCP: Endoscopic retrograde cholangiopancreatography
PEP: Post-ERCP pancreatitis
RCT: Randomized controlled trial
SOD: Sphincter of Oddi
CBD: Common bile duct
NKF: Needle-knife fistulotomy
NKS: Needle-knife sphincterotomy
TPS: Trans-pancreatic precut sphincterotomy
SPIRIT: Standard Protocol Items: Recommendations for Interventional Trials
LMWH: Low molecular weight heparin
DOAC: Direct-acting oral anticoagulant
KHSC: Kingston Health Sciences Centre
